# Randall-Type Monoclonal Immunoglobulin Deposition Disease: New Insights into the Pathogenesis, Diagnosis and Management

**DOI:** 10.3390/diagnostics11030420

**Published:** 2021-03-02

**Authors:** Camille Cohen, Florent Joly, Audrey Sibille, Vincent Javaugue, Estelle Desport, Jean-Michel Goujon, Guy Touchard, Jean-Paul Fermand, Christophe Sirac, Frank Bridoux

**Affiliations:** 1 Department of Nephrology Hôpital Necker, and INSERM U830 “Stress and Cancer” Laboratory, Institut Curie, 75015 Paris, France; 2 Department of Nephrology, CHU Poitiers, 86000 Poitiers, France; joly.floren@gmail.com (F.J.); audrey.sibille@chu-poitiers.fr (A.S.); vincent.javaugue@chu-poitiers.fr (V.J.); estelle.desport@chu-poitiers.fr (E.D.); Guy.TOUCHARD@chu-poitiers.fr (G.T.); Frank.BRIDOUX@chu-poitiers.fr (F.B.); 3 Centre National de Référence Maladies Rares: Amylose AL et Autres Maladies à Dépôts d’Immunoglobulines Monoclonales, 86000 Poitiers, France; 4 INSERM CIC 1402, 86000 Poitiers, France; 5 CNRS UMR 7276-CRIBL, University of Limoges, 87000 Limoges, France; christophe.sirac@unilim.fr; 6 Department of Pathology, CHU Poitiers, 86000 Poitiers, France; Jean-Michel.GOUJON@chu-poitiers.fr; 7 Department of Immunology and Hematology, Hôpital Saint Louis, 75010 Paris, France; jpfermand@yahoo.fr

**Keywords:** MGRS, plasma cell dyscrasia, glomerular disease, monoclonal gammopathy

## Abstract

Randall-type monoclonal immunoglobulin deposition disease (MIDD) is a rare disease that belongs to the spectrum of monoclonal gammopathy of renal significance (MGRS). Renal involvement is prominent in MIDD, but extra-renal manifestations can be present and may affect global prognosis. Recent data highlighted the central role of molecular characteristics of nephrotoxic monoclonal immunoglobulins in the pathophysiology of MIDD, and the importance of serum free light chain monitoring in the diagnosis and follow-up disease. Clone-targeted therapy is required to improve the overall and renal survival, and the achievement of a rapid and deep hematological response is the goal of therapy. This review will focus on the recent progress in the pathogenesis and management of this rare disease.

## 1. Introduction

Monoclonal immunoglobulin deposition disease (MIDD) is defined by the linear deposition of monoclonal immunoglobulin (MIg) along renal basement membranes [1]. According to the composition of the deposits, MIDD can be subcategorized as light chain deposition disease (LCDD), heavy chain deposition disease (HCDD), or light and heavy chain deposition disease (LHCDD) [1,2,3,4,5,6]. 

MIDD is a systemic disorder with predominant renal manifestations, but other organs can be involved, such as the heart or the liver, although less frequently than in AL amyloidosis. MIDD often presents with progressive kidney failure associated with glomerular proteinuria and/or hematuria, and sometimes as isolated slowly progressive kidney failure [1,2,3,7].

Although linear tissue deposition of monoclonal LC was first reported in the 1950s, LCDD was fully described by Randall et al. in 1976 [8,9,10], LHCDD and HCDD being later identified in the 1990s [11,12]. These three categories were included within the spectrum of renal lesions associated with gammopathy of renal significance (MGRS). The concept of MGRS was introduced in 2012 to describe small B-cell clonal disorders responsible for renal lesions, not related to the tumor burden but to the production of nephrotoxic monoclonal immunoglobulins [13]. Indeed, even if MIDD can be observed in the context of symptomatic myeloma (or less commonly of a lymphoid neoplasm), it is predominantly associated with an otherwise asymptomatic plasma cell disorder. The concept of MGRS not only highlights the toxic and possibly life-threatening effects of the monoclonal immunoglobulin, but it also justifies the use of clone-directed therapy, the sole efficient strategy currently available to preserve renal and patient outcomes. 

As MIDD is a rare disease, its prevalence and incidence are unknown. MIDD is thought to represent less than 0.1% of diagnoses on native kidneys. Recently, several large studies have brought important advances on the comprehension and management of the disease. 

## 2. Pathophysiology

Histologically, the hallmark of MIDD, whatever its type, is the presence of linear amorphous monoclonal immunoglobulin deposits along basement membranes, particularly in the kidney. In contrast to renal AL amyloidosis, which is characterized by deposition of straight, unbranched fibrils that predominate in the mesangium, MIDD deposits display a finely granular powdery punctuate ultrastructural appearance and are mainly distributed in the outer part of the renal tubular basement membrane, and on the inner part of the glomerular basement membrane. Another striking characteristic of MIDD is the accumulation of extracellular matrix (ECM) in the glomerulus, participating in the progressive development of glomerular damage. These specific characteristics of MIDD suggest that the involved MIg have peculiar physicochemical characteristics. 

### 2.1. LCDD

Recent experimental studies and the development of mouse models have helped deciphering the importance of the MIg structure in the pathogenesis of MIDD. The injection to mice of a murine hybridoma expressing a pathogenic human Vk4 light chain isolated from a patient with LCDD recapitulated the multisystemic linear deposits of light chains [14]. Although the sequencing of a large number of LC from LCDD patients have highlighted the overrepresentation of some kappa LC subtypes, such as Vκ1 (IGKV1-5), Vκ3 (IGKV3-11 and IGKV3-15), or Vκ4 (IGKV4-1) subgroups [15,16], no redundant mutations have been identified. Nevertheless, several abnormalities have been observed. Hydrophobic residues at unusual positions or abnormal N-glycosylation sites in variable domains are common, probably favoring LC aggregation [17,18,19]. Recent data further suggest that modification of the isoelectric point could be the main driver of LC deposition. In contrast with AL amyloidosis-prone monoclonal LC in which isoelectric point is highly variable, variable domain of LCDD LC almost always present a cationic isoelectric point, above 7.5 [20,21]. This means that at physiological pH, these LC are positively charged, and could electrostatically interact with the outer part of the tubular basement membrane (TBM), and the inner part of the glomerular basement membrane (GBM), both charged negatively. 

Recently, a mouse model of LCDD provided further insights into the mechanisms of LCDD. Bender et al. developed a transgenic model using site-directed insertion of the variable domain of a pathogenic human VK4 LC gene into the mouse immunoglobulin kappa locus, ensuring the production by all plasma cells (PCs) of a hybrid LC composed of the human V domain and the murine constant domain [22]. This mouse model recapitulates major hallmarks of MIDD, including progressive glomerulosclerosis, nephrotic-range proteinuria, and finally, kidney failure. It also confirms that pathogenic properties of LCDD light chains are entirely bore by the variable domain. 

Kidney lesions are similar to those observed in humans, featuring the progressive development of nodular glomerulosclerosis resulting in proteinuria and kidney failure. These lesions are severe, leading to the death of mice within a median of 8.5 months. One major point of this study is that PC expressing the pathogenic LC exhibit a high level of endoplasmic reticulum stress, supporting the use of proteasome inhibitors in LCDD. Interestingly, regression of kidney lesions was observed in mice treated with proteasome inhibitors. 

The molecular mechanisms underlying the development of glomerulosclerosis in MIDD are still unknown. In vitro studies have shown that the stimulation of mesangial cells with pathogenic light chains isolated from MIDD patients resulted in increased TGFβ-induced secretion of ECM proteins, particularly collagen IV, laminin and tenascin [23]. Moreover, mesangial cells incubated with LCDD LC undergo a myofibroblast-like phenotypic transformation [24]. Transcriptomic analysis of pre-sclerotic glomeruli, performed in the model developed by Bender at al., identified that two main pathways, i.e., cellular proliferation and ECM remodeling, are activated after interaction of pathogenic light chains with mesangial cells [22]. Work is in progress to explore the precise molecular mechanisms leading to glomerular morphologic alterations in LCDD. The sequence leading from the deposit to the expansion of extracellular matrix is not yet characterized. Whether it is only the mesangial cells that are involved in this expansion still needs to be explored. On the same line, the molecular events occurring into the mesangial cells that are responsible for matrix accumulation are to be defined. The understanding of these molecular mechanisms could help in the development of targeted therapy, avoiding the use of chemotherapy in this disease. 

### 2.2. Structural Peculiarities in HCDD

At the opposite of LCDD, monoclonal heavy chains in HCDD present an invariable characteristic, i.e., deletion of their first constant domain (CH1) [25]. Physiologically, during the synthesis of Ig, CH1 interacts with the BIP chaperon in the endoplasmic reticulum of plasma cells to avoid the release of free heavy chains. If CH1 is deleted, BIP cannot associate to CH1 and free heavy chains can be secreted and deposited along basement membranes. Of note, despite the absence of detection of MIg by conventional techniques in 40 to 50% of cases, all HCDD patients present with an abnormal serum free light chain ratio [21,25,26]. Recently, immunofluorescence studies of bone marrow from HCDD patients revealed that clonal PC secrete both the pathogenic truncated HC and a non-toxic free LC. Although this LC does not deposit in tissues, it can be used as a surrogate marker of HC secretion and of the underlying clone activity [27]. A mouse model of HCDD has been generated by knock-in insertion of a human HC in the mouse kappa LC locus. Conditional deletion of CH1 [28] was followed by HC deposition, reproducing the cardinal pathologic feature of human HCDD. As in LCDD, the authors showed that PCs secreting the HC experience high level of ER stress, and display high sensitivity to proteasome inhibition [28]. Strikingly, truncated HC, as LCDD-prone LC, are also characterized by high isoelectric points of variable domains [25,28]. Nevertheless, in this model, mice do not develop nodular glomerulosclerosis, even after prolonged follow-up. This suggests that CH1 deletion is essential for the secretion and the deposition of HC but might not be sufficient for the development of glomerular injury. The nephrotoxic properties of monoclonal HC appear restricted to the variable domain. Indeed, in heavy chain disease, a different lymphoplasma cell proliferative disorder, in which no organ deposits are observed, a deletion of the CH1 domain is also found. Nevertheless, it is always associated with a partial or complete deletion of the V domain which probably explains the absence of deposits [29].

Finally, very few data exist about LHCDD, but the disease probably derives from the simultaneous secretion of both a pathogenic LC and truncated heavy chain by the same clone [30].

## 3. Diagnosis

MIDD is a rare disease, more frequently observed in middle-aged men [1,2,3,21,26,31,32,33]. Clinical presentation can be variable, as highlighted by several recent studies. 

### 3.1. Renal Involvement

Renal involvement is prominent in virtually all patients with MIDD. It manifests usually with chronic glomerular symptoms, with nearly constant proteinuria and nephrotic syndrome in 20 to 60% of cases [21,31,32,33]. Contrasting with AL amyloidosis, microhematuria and hypertension are frequent, present at diagnosis in up to 75% of patients [21,31,32,33]. It is worth noting that all subtypes of MIDD do not share the same clinical features. HCDD patients present more frequently with nephrotic syndrome than LCDD [21]. On the other hand, LCDD can present under three major phenotypes. First, pure LCDD, the most common, usually manifests as a glomerular disease with severe chronic kidney disease (stage 3 or higher). Second, LCDD can present with progressive CKD, and mild or absent proteinuria (less than 0.5 g/day) [7]. This subcategory is probably underrecognized, as patients without proteinuria less frequently undergo kidney biopsy. This subtype is characterized by severe vascular lesions and extensive interstitial fibrosis on the kidney biopsy [7,21]. Finally, in patients with symptomatic myeloma, LCDD may coexist with myeloma cast nephropathy. In these cases, the presentation is close to that of myeloma cast nephropathy, except for higher level of albuminuria. Histologically, linear peritubular and glomerular deposits are sometimes detected by IF only, and the associated characteristic light microscopy and ultrastructural features of the disease are frequently lacking. The prognosis and management of LCDD-associated LC cast nephropathy are identical to that of MCN. 

### 3.2. Extra-Renal Involvement

**Figure 1:**
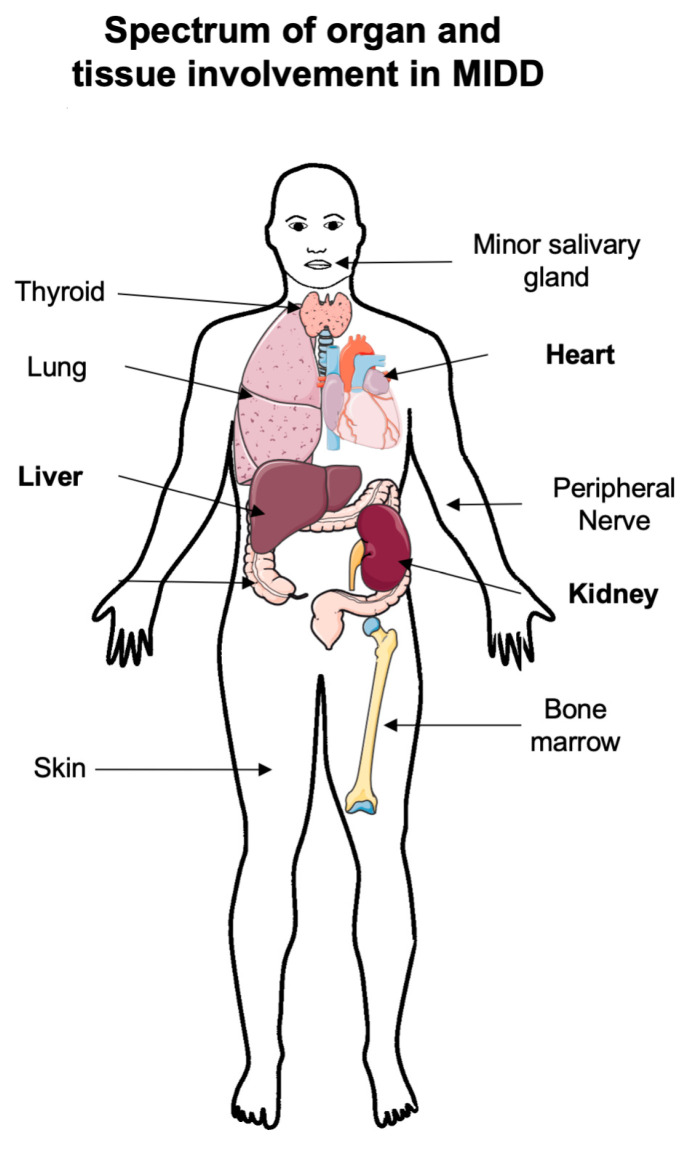
Schematic representation of extra-renal involvement in MIDD (monoclonal immunoglobulin deposition disease). Adapted from Joly, Cohen et al. [21] Randall-type monoclonal immunoglobulin deposition disease: novel insights from a nationwide cohort study. Blood.

MIDD is a systemic disease, but compared to AL amyloidosis, extra-renal involvement (Figure 1) is often pauci-symptomatic. In a recent nationwide cohort, up to 50% of patients with pure LCDD had at least one extra-renal manifestation [21]. Systemic manifestations are less common in HCDD. 

Liver involvement occurs in up to 20% of cases, mainly characterized by isolated elevation in liver enzymes without hepatocellular insufficiency [21]. Rarely, it can lead to fulminant hepatitis [34]. 

Cardiac involvement has been reported in around one third of LCDD patients [35,36]. The most common symptoms are dyspnea and arrhythmias or conduction disorders, including atrial fibrillation, prolonged QT interval or sinus bradycardia. Doppler echocardiography shows hypertrophic cardiomyopathy resembling AL amyloidosis heart disease, with increased septal thickness and diastolic dysfunction, usually with preserved left ventricular ejection fraction [21,35]. As in AL amyloidosis, BNP/NT-proBNP and troponin are early markers of cardiopathy, but their prognostic value is still unclear [21,35]. Even though heart disease in LCDD appears less severe than that of AL amyloidosis, it has been reported as a major risk factor for mortality after autologous stem cell transplantation [35]. 

MIDD deposits have been described in virtually all tissues and organs, including central or peripheral nervous system, gastrointestinal tract, pancreas, adrenal glands, lung, thyroid, eye and salivary glands [5,21,37]. 

Cystic lung disease is a rare form of localized LCDD, without other organ involvement. It typically affects young adults, and manifests with progressive obstructive pulmonary disease, with numerous cysts diffusely distributed in both lungs and emphysematous-like lesions. Rapid progression to severe respiratory insufficiency is common. Histologically, it is characterized by linear amorphous deposits of monoclonal LC in the alveolar walls, small airways and vessels, produced by a locally infiltrating intrapulmonary B-cell clone. Cystic lung disease appears to be specifically associated with the VK1-8 LC subgroup. When feasible, lung transplantation is an efficient therapeutic option, which, by eliminating the local clone results in satisfactory long-term survival, without disease recurrence [38,39,40].

## 4. Immunologic and Hematologic Characteristics

By definition, the presence of renal monotypic immunoglobulin deposits implies the presence of an underlying plasmacytic or lymphoplasmacytic clone. Historically, MIDD has been associated to multiple myeloma in around 50% of cases, a frequency that varied with the definition used for the diagnosis of multiple myeloma [1,2,3]. Recent series, using current diagnostic criteria for myeloma, have established that 60 to 80% of MIDD are associated with an indolent clone, corresponding to MGRS [21,30,31,32,33]. 

The identification of the nature of the clone, i.e., composed of plasma cells or B-lymphocytes, is essential for the choice of an adapted efficient chemotherapy. Thus, careful hematologic and immunologic workup is mandatory for the management of MIDD. In historic studies, 15 to 30% of patients failed to display a detectable monoclonal gammopathy by conventional techniques, i.e., serum electrophoresis and immunofixation [1,2,3]. Interestingly, and contrasting with AL amyloidosis, recent studies have highlighted that virtually all patients with MIDD have an abnormal serum free light chain level and ratio at diagnosis [21,26]. Serum free light chain measurement should therefore be performed in every patient with a suspicion of MGRS. Besides diagnostic considerations, pre-treatment serum FLC (free light chain) level is also required to evaluate and quantify hematologic response after chemotherapy. As described above, LCDD can present without proteinuria, and may be misdiagnosed as nephroangiosclerosis or ischemic nephropathy, particularly in older patients. In this situation, the finding of an abnormal κ/λ ratio is suggestive of MGRS, and should prompt considering hematologic investigations and kidney biopsy [41]. 

Hematological workup should be as complete as possible and include bone marrow studies, with flow cytometry to detect a subtle clone, most commonly composed of plasma cells in MIDD, and cytogenetic studies. More sensitive techniques, such as next generation sequencing (NGS) are increasingly available and will probably increase the clone detection rate in the near future [42]. Flow cytometry of peripheral B cells should be performed if bone marrow studies are inconclusive, since MIDD is sometimes associated with non-Hodgkin B-cell lymphoma [21]. Imaging studies, including X-rays, low-dose whole body-CT scan, or MRI, PET-scan or PET-MRI should be performed to identify lytic bone lesions or solitary plasmacytomas [4]. 

## 5. Pathology

**Figure 2:**
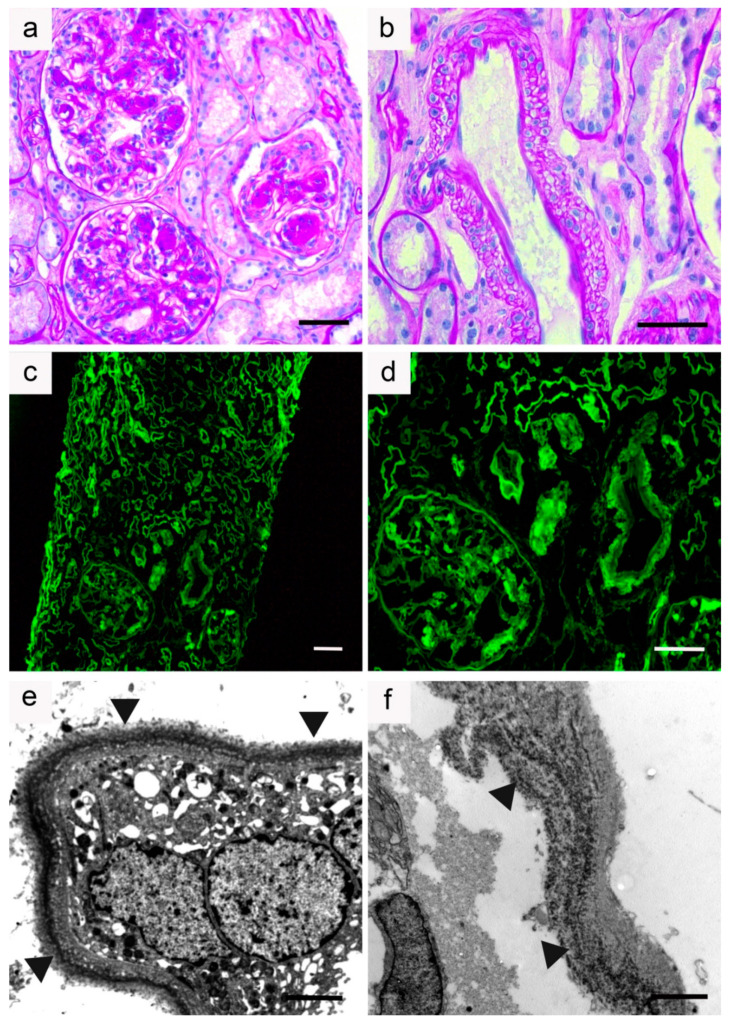
Kidney biopsy sample from a patient with κ-light chain deposition disease: (**a**,**b**) Light microscopy, periodic acid-Schiff staining (PAS); (**a**) Section of renal cortex showing nodular glomerulosclerosis with nodular mesangial deposits and aneurysmal dilatation of the capillary lumens (×200); (**b**) Note the presence of PAS-positive deposits along vascular myocytes and tubular basement membranes (×400). Bar = 50 µm; (**c**,**d**) Immunofluorescence microscopy (anti-κ conjugate). Glomerular deposits and linear deposits along tubular basement membranes and vascular myocytes (**c**: ×100 and **d**: ×200). Bar = 50 µm; (**e**,**f**) Electron microscopy. (**e**) Enlarged tubular basement membrane with electron-dense powdery punctuate deposits (arrows) predominating in the outer aspect (×15,000). Bar = 2 µm; (**f**) Linear electron-dense deposits (arrows) predominating along the endothelial inner aspect of the glomerular basement membrane (×12,000). Bar = 2 µm.

**Figure 3:**
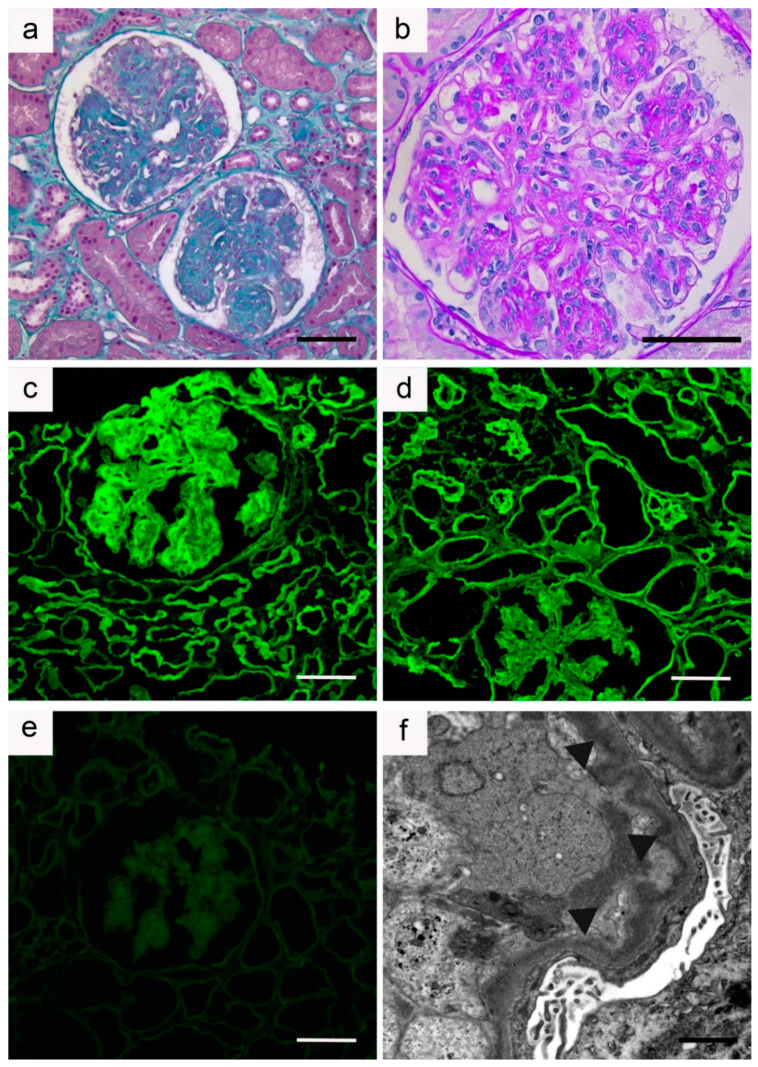
Kidney biopsy sample from a patient with γ1-heavy chain deposition disease: (**a**,**b**) Light microscopy; (**a**) Section of renal cortex showing nodular glomerulosclerosis (Masson’s trichrome staining: ×200); (**b**) Nodular glomerulosclerosis with diffuse mesangial nodular periodic acid-Schiff (PAS) positive deposits, mesangiolysis and moderate mesangial hypercellularity (PAS staining: ×400). Bar = 50 µm; (**c**–**e**) Immunofluorescence microscopy (×200). Positive staining in the mesangium and along glomerular and tubular basement membranes with anti-γ (**c**) and anti-γ1 conjugates (**d**). No significant staining was observed using anti-γCH1 antibody (**e**) suggestive of a first constant domain (CH1) deletion. Bar = 50 µm; (**f**) Electron microscopy (×12,000). Linear electron dense deposits (arrows) predominating along the inner aspect of the glomerular basement membrane. Bar = 2 µm.

Although MIDD commonly manifests with predominant glomerular symptoms, tubular involvement is constant. By light microscopy, ribbon-like thickening of tubular basement membranes with PAS positive deposits is observed in most cases. Glomerular lesions are frequent and predominate in the mesangium, with increased extracellular matrix admixed with Congo-red negative PAS-positive deposits. They ultimately produce a characteristic pattern of nodular glomerulosclerosis, observed in approximatively 60% of LCDD cases and in virtually all HCDD cases [21,26,32]. Pseudo aneurysmal dilatation of glomerular capillaries is common. Glomerulosclerosis in MIDD may be distinguished from Kimmelstiel-Wilson nodules in diabetic nephropathy, by the bitonal appearance of lesions (composed of MIg and ECM proteins) and their lower intensity with methamine silver-staining. Glomerular crescents are rare, except in alpha HCDD, whereas arteriolar lesions are common. Variable degrees of tubular atrophy, interstitial fibrosis, and inflammation may be seen. Immunofluorescence studies are mandatory to confirm the diagnosis of MIDD, showing diffuse linear staining along basement membranes of glomeruli, tubules, and around vascular myocytes for a single LC isotype (most commonly kappa) in LCDD, for a single Ig heavy chain and LC in LHCDD, or for a single class of Ig with CH1 domain deletion and no corresponding LC in HCDD. Ultrastructural examination typically reveals punctate ‘powdery’ electron-dense deposits along the inner aspect of glomerular basement membranes and outer aspect of tubular basement membranes (Figure 2 and Figure 3). 

In patients with MIDD and symptomatic multiple myeloma, LCDD (often without nodular glomerulosclerosis) frequently coexist with LC cast nephropathy [21,43]. In this setting, deposits may not be detected by electron microscopy, a condition referred to as “LCDD by IF only” [1]. Concurrent LCDD and amyloid deposits composed of the same LC isotype has been sometimes reported [44,45].

## 6. Response Criteria

As described above, the obtention of a hematologic response is mandatory to improve renal and global outcomes in MIDD patients. In the absence of specific response criteria, the studies published to date have used the hematologic response criteria developed in 2012 by the International Society for Amyloidosis (ISA) for AL amyloidosis [46]. These criteria are based on the difference between the involved and the uninvolved FLC (dFLC). (Table 1). Complete hematologic response (CR), very good partial response (VGPR) and partial response are defined by normalization of FLC with negative serum immunofixation (CR); achievement of dFLC <40 mg/L (VGPR); ≥50% decrease in dFLC (PR). Renal response is defined by a 50% decrease in proteinuria (baseline proteinuria must be >0.5 g/day) without ≥25% decrease in baseline eGFR value. Nevertheless, these criteria remain to be validated in MIDD. Currently, the International Kidney and Monoclonal Gammopathy Research Group (IKMG) is conducting a multicentric international study to define specific hematologic and renal response criteria for LCDD. 

## 7. Treatment and Prognosis

### 7.1. Chemotherapy

In historical series, published before the era of novel anti-myeloma agents, the prognosis of MIDD was poor, with a median renal survival of two years and a median patient survival of four years [1,2]. 

The first evidence of the strong correlation between hematological response and renal outcomes was provided by Royer et al. in 2004. In this study, 11 young and fit MIDD patients received high dose melphalan followed by autologous stem cell transplant (HDM/ASCT) [47]. This treatment was well tolerated despite impaired renal function, with acceptable tolerance contrasting with the increased morbidity and mortality associated with this procedure in patients with multiple myeloma and renal insufficiency. [48,49]. Hematological response, based on the M-spike decrease, was obtained in 70% of patients, whereas renal response was achieved in around 30% of cases [47]. 

Recently, several studies have validated the efficacy of proteasome inhibitor-based regimens in MIDD, and the value of serial serum free light chain measurements for evaluating hematological response. Furthermore, they showed that criteria defining hematological response in AL amyloidosis can be used in MIDD, with the same predictive value for long-term outcomes. These response criteria are recapitulated in Table 1. In a French series, among 49 patients treated with bortezomib-containing combinations, 78% achieved very good partial response (VGPR) or complete response (CR). Renal response was obtained in 53% of patients, with a 35% median improvement in eGFR value [26]. Interestingly, the sole factor independently associated with renal response was hematological response based on FLC levels. Several other studies confirmed that proteasome inhibitors are well tolerated, and allow high rates of deep hematologic and renal responses [31,32,33]. Results of these studies are recapitulated in Table 2. 

Recently, a large French study, based on 255 patients [21] identified several factors independently associated with renal response, such as achievement of at least a very good partial hematological response, and the absence of severe interstitial fibrosis on diagnostic kidney biopsy. Deep hematologic response was also independently associated with overall survival. Of note, hematologic remissions, usually sustained, were more frequent in patients treated with proteasome inhibitor-based combinations, which currently appear as a safe and efficient first-line strategy in MIDD. 

The place of HDM/ASCT in therapeutic strategy is still to be clarified. In the different studies published to date, HDM/ASCT induced high rate of hematologic and renal responses. Nevertheless, whether HDM/ASCT is superior to bortezomib-based regimen is unclear. Indeed, MIDD patients who received HDM/ASCT were younger, with better renal function, making it difficult to compare between these two strategies [21,26,47]. 

Recently, targeted anti-plasma cell therapy using anti-CD38 monoclonal antibodies has shown excellent efficacy and tolerance profile in multiple myeloma and AL amyloidosis. It is likely that these agents will rapidly play a major role in the treatment armamentarium of MIDD [50,51,52]. Recently, in a series of 8 patients with LCDD and multiple myeloma treated with the anti-CD38 monoclonal antibody daratumumab because of hematologic relapse, hematologic response was obtained in 7/8 patients, with stabilization of renal function [53]. Further studies are needed to refine the therapeutic management of MIDD, and particularly to assess the place of anti-CD38 monoclonal-based regimens as first-line therapy. 

### 7.2. Symptomatic and Supportive Measures

As in every chronic kidney disease, therapies to limit kidney disease progression must be introduced, according to current recommendations [54]. Blockers of the renin angiotensin system should be used to reduce proteinuria and to control hypertension, ideally to maintain blood pressure below 130/80 mmHg. 

### 7.3. Renal Transplantation for MIDD

Until recently, renal transplantation was considered a poor option in MIDD, due to concerns regarding disease relapse on the allograft, and the risk of progression to symptomatic malignant hematological disease. In a case series published in 2004, before the era of novel anti-myeloma agents and free light chain measurements, among 7 MIDD patients who received a renal transplant, relapse on the allograft was observed in 5, and death occurred in 4 after a median of 12 years after transplantation [55]. Four had not received any hematological treatment prior to transplantation and none had achieved a deep hematologic response. In a recent study, 23 patients with MIDD received a kidney transplant. Of the 14 patients who had obtained deep hematologic response before renal transplantation, all had a functional kidney allograft after a median follow-up of more than 7 years. Disease recurrence occurred in 4, but was successfully treated with anti-plasma cell agents [21]. These preliminary results suggest that, as in AL amyloidosis, renal transplantation can be offered to selected MIDD patients, with acceptable long-term results, provided deep and sustained hematological remission is achieved before the procedure [21]. 

## 8. Treatment Recommendations

**Figure 4:**
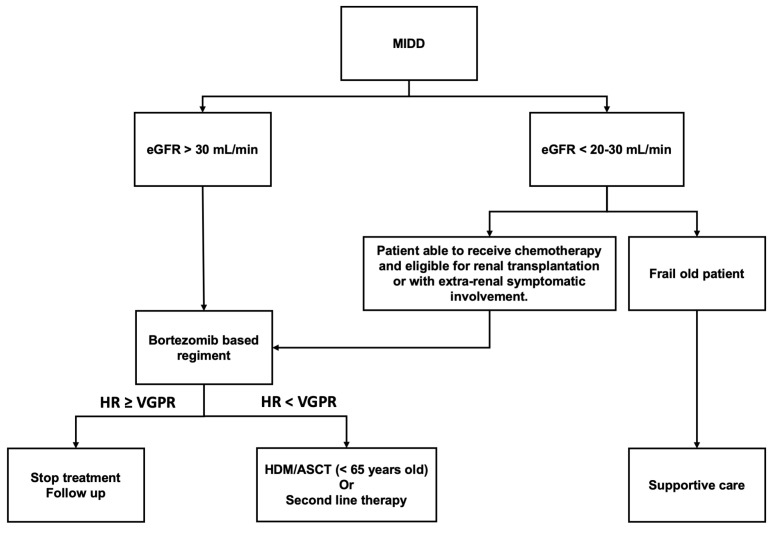
Proposed algorithm for treatment decisions in MIDD.

Patients who are fit enough to receive treatment and with eGFR > 20 or 30 mL/min/1.73 m^2^ or symptomatic extra-renal involvement should receive clone-targeted chemotherapy, using a bortezomib-based regimen in those with an underlying plasma cell clone. The goal of therapy is to rapidly obtain a deep hematologic response, i.e., VGPR or above. In young patients, HDM/ASCT could be considered in case of insufficient hematologic response, particularly if eGFR is ≥ 30 mL/min/1.73 m^2^. Patients with low eGFR (<20 mL/min/1.73 m^2^) and severe interstitial fibrosis on kidney biopsy should be offered chemotherapy only if a renal transplantation is considered, or if symptomatic extra-renal involvement is present. For elderly patients with renal-limited disease, and those with impaired performance status, chemotherapy is generally not indicated, and treatment should be discussed in each individual case. Treatment algorithm is illustrated in Figure 4.

## 9. Conclusions

MIDD are rare and heterogeneous systemic diseases, caused by systemic deposition of a pathogenic monoclonal immunoglobulin, with prominent renal involvement. MIDD always complicates an underlying B-cell clonal disorder, more frequently plasmacytic, that usually corresponds to MGRS and less frequently to an overt multiple myeloma or B-cell lymphoma. 

Recent studies have underlined several major characteristics of MIDD. First, serum FLC level is elevated in nearly all patients with MIDD, including those with HCDD. Besides their diagnostic value, serum FLC assays should be regularly performed throughout the disease evolution to assess hematologic response, which is the goal of therapy, and to detect early relapse. As in AL amyloidosis, the quality of hematologic response is the main prognostic factor for long-term renal and patient survival. Early diagnosis with prompt initiation of clone-targeted chemotherapy with bortezomib-based regimens is the current most efficient strategy in MIDD. In the near future, management of MIDD is likely to be facilitated through the definition of international response criteria and the use of novel anti-plasma cell agents, particularly anti-CD38 monoclonal antibodies in which efficacy and good tolerance profile have been established in multiple myeloma. As in other types of MGRS-related renal disorders, a multidisciplinary approach with close collaboration between hematologists and nephrologists is required. 

## Figures and Tables

**Table 1:** International Society of Amyloidosis criteria for hematologic and renal responses in AL amyloidosis. Adapted from Palladini et al. [46].

*Complete Response (CR)*	Negative Serum and Urine Immunofixation Normal FLC Ratio
*Very good partial response (VGPR)*	dFLC < 40 mg/L
*Partial response (PR)*	>50% decrease in dFLC
*No response (NR)*	None of the above criteria

dFLC: difference between the involved and the uninvolved free light chain (FLC).

**Table 2:** Summary of the main recent studies on MIDD.

	Nasr et al. [3]	Cohen et al. [26]	Kourelis et al. [31]	Sayed et al. [32]	Ziogas et al. [33]	Joly et al. [21]
Period of the study	1992–2011	2005–2013	1992–2014	2002–2015	2005–2015	1981–2015
Number of patients	64	49	88	53	18	255
MIDD type (n)	LCDD (51), HCDD (7), LHCDD (6)	LCDD (35), HCDD (12), LHCDD (2)	LCDD (74), HCDD (7), LHCDD (7)	LCDD (53)	LCDD (14), HCDD (3), LHCDD (1)	LCDD (212), HCDD (23), LHCDD (20)
Follow-up (months)	25	54	47	74	39	27.3
ESRD during follow-up (%)	39	20.4	33	53	33.3	40.3%
Death during follow-up (%)	32	10	38	36	28	33
Factors associated with renal response by multivariate analysis	Serum creatinine at diagnosis	dFLC < 40 mg/L after treatment	eGFR > 20 mL/min/1.73 m^2^ VGPR/CR Renal response	Not performed	Non-significant	VGPR/CR Absence of severe interstitial fibrosis

Abbreviations: LCDD—light chain deposition disease; HCDD—heavy chain deposition disease; LHCDD—light and heavy chain deposition disease; ESRD—end stage renal disease; VGPR/CR—very good partial response/complete response; eGFR—estimated glomerular filtration rate.
